# Prescribing practices of corticosteroids in outpatient dermatology department of Injibara General Hospital, north-West Ethiopia, 2024

**DOI:** 10.3389/fmed.2024.1496784

**Published:** 2025-01-03

**Authors:** Alemu Bezabih Tegegnie, Tamiru Alene, Workineh Tamir, Meaza Molla Sineshaw

**Affiliations:** ^1^Department of Dermatovenereology, College of Medicine and Health Sciences, Injibara University, Injibara, Ethiopia; ^2^Department of Pediatrics and Child Health Nursing, College of Medicine and Health Sciences, Injibara University, Injibara, Ethiopia; ^3^Department of Medical Laboratory Science, College of Medicine and Health Sciences, Injibara University, Injibara, Ethiopia; ^4^Public Health Officer, Ethiopian Statistical Service, Bahirdar, Ethiopia

**Keywords:** corticosteroids, prescribing practice, dermatology, Injibara, Ethiopia

## Abstract

**Background:**

Corticosteroids are among the most frequently prescribed drugs in the world because they are extremely effective for the relief of symptoms of many inflammatory and immune disorders and other conditions. Corticosteroids have been a mainstay of pharmacotherapy in dermatological practice.

**Objective:**

This study aimed to assess prescribing practices of corticosteroids in outpatient dermatology department of Injibara General Hospital, North-West Ethiopia, 2024.

**Method:**

A facility-based retrospective cross-sectional study was conducted from September 1 to September 10, 2024, with 422 patient prescriptions issued at the dermatology outpatient department of Injibara General Hospital containing at least one corticosteroid medicine. All patient prescriptions dispensed from the dermatology outpatient department from April to August 2024 containing at least one corticosteroid medicine were included. A structured data collection tool was used to collect data, and Statistical Package for Social Science version 27.1 was used for data analysis. The study population was characterized through descriptive analysis.

**Results:**

Female patients accounted 54.2% of the cases recorded, and the most common age group was between 21 and 30 years (26.5%). Atopic dermatitis (15.4%) was the most common skin disorder, followed by papular urticaria (9.97%) and seborrheic dermatitis (8.9%) for which corticosteroids were prescribed. Topical corticosteroids were the most commonly prescribed medications, accounting for 94.1%. The percentage of corticosteroids prescribed by generic name was 82.4%. We found that the percentage of topical corticosteroid prescriptions in fixed drug combinations and extemporaneous prescription was 4.4 and 1.2%, respectively. Mometasone furoate was the most commonly prescribed (34.6%), followed by clobetasol propionate (26.7%). Ointments (53%) followed by creams (40.6%) were the most common formulations of topical corticosteroids issued. In the present study, moderate potency TCS were most commonly prescribed (32.3%), followed by high potency (28.3%), and super high potency (27.6%), respectively.

**Conclusion:**

From this study, it can be concluded that atopic dermatitis is the most common skin disease observed in the studied dermatology outpatient where the corticosteroid was indicated. Mometasone furoate and clobetasol propionate were the two most commonly prescribed agents. Prescribing practice of high potent and very high potent topical corticosteroids was found to be considerably high.

## Introduction

Skin is part of the integumentary system and is the largest organ of the human body (1). Skin diseases refer to disorders predominantly affecting the layers of the skin, including the epidermis, dermis, and subcutaneous tissue (2). Dermatological disorders are among the leading causes of hospital visit in Ethiopia accounting for 25% of outpatient Department (OPD) cases (3).

Steroids are among the most frequently prescribed drugs in the world because they are extremely effective for the relief of symptoms of many inflammatory and immune disorders and other conditions (4). Corticosteroids have been a mainstay of pharmacotherapy in dermatological practice (5). According to the severity of the underlying condition, anatomic location of application and patient age, corticosteroids of different potencies are prescribed either in topical or systemic routes (6). The amount and potency of corticosteroid which is prescribed, dispensed and applied should be considered carefully (7). Since the introduction in early 1950s, topical corticosteroids (TCs) have become the most commonly prescribed drugs by dermatologists in an outpatient setting (8).

The present study aims to evaluate prescribing practices of corticosteroids in outpatient dermatology department among patients visiting dermatology OPD.

## Methods

### Study setting and period

This study was conducted from September 1 to September 10, 2024, at Injibara General Hospital, Northwest Injibara, the capital of the Awi zone, is expected to have 40,836 inhabitants in 2024 (9). The city currently has one health center and two General hospitals (i.e., one public and one private hospital). Injibara General Hospital started providing dermatological services in April 2024.

### Study design

A facility-based retrospective cross-sectional study was conducted.

### Population characteristics

#### Source population

All patient prescriptions issued at the dermatology OPD of Injibara General Hospital were considered as a source population.

#### Study population

All patient prescriptions dispensed from April to August 2024 at the dermatology OPD of Injibara General Hospital containing at least one corticosteroid medicine constitute the study population.

### Inclusion and exclusion criteria

All patient prescriptions issued at the dermatology OPD of Injibara General Hospital containing at least one corticosteroid medicine during the study period will be included. Those prescriptions that were legible and complete were included.

### Sample size determination and sampling procedure

The sample size was maximized using the proportions of corticosteroid prescription pattern (*p* = 0.5) for unknown prevalence. The margin of error (d) of 5% was used with a 95% confidence interval. With a 10% contingency for incomplete or lost prescription papers, the final sample was 422. From April to August 2024, a total of 1250 prescriptions that were dispensed in the hospital outpatient pharmacy were found. Out of this, 417 prescriptions contained at least one corticosteroid. As a result, we included all patient prescriptions issued at dermatology OPD of Injibara General Hospital containing at least one corticosteroid medicine during this time period to satisfy our estimated sample size.

### Method of data collection

First, the principal investigator gave a half-day of training on data collection and management to a supervisor and data collectors on September 1, 2024. From September 3 to September 10, 2024, a structured data collection tool was used to collect data. It was employed after it was translated into the Amharic version by a linguist to collect the relevant data from the patient prescription paper. The source of data was the hospital outpatient pharmacy. The socio-demographic information (age and sex) was presented in the first section. The second part comprises patterns of skin diseases. The last part includes patterns of corticosteroid drug prescription at the dermatology OPD of Injibara General Hospital. Two trained nurses collected the data.

### Operational definitions and measurements of variables

Fixed Drug Combinations describes a combination of two or more medicinal products packaged together.

Extemporaneous compounding describes the use of traditional compounding techniques to manipulate chemical ingredients to produce appropriate dosage forms when no commercial medicines form is available (10).

### Data processing and analysis

Each item of data was coded and assigned a special identification number. A different value than the potential replies was assigned to the missing value. Duplicate entries were eliminated. Cross -tabulation was used to verify the answers’ logical coherence. If a coding error exists, data is erased if it cannot be retrieved or logically fixed. The cleaned and edited data were ready for appropriate statistical analysis.

The data collected using a structured data collection tool was entered into Epi-data version 3.1 and exported and analyzed using Statistical Package for Social Science Studies (SPSS) version 27.1. A descriptive analysis using frequency and percentage of the variables was done. The result of the analysis was presented using tables and chart.

## Results

### Socio-demographic characteristics

A total of 404 prescriptions were evaluated, yielding a response rate of 95.7%. Two hundred nineteen (54.2%) of the cases recorded were female, and the most common age group was between 21 and 30 years (26.5%). See Table 1.

**Table 1:** Age with sex distribution of patients attending dermatology OPD, Injibara General Hospital, 2024.

Age (years)	Male, n (%)	Female, n (%)	Total, n (%)
0–10	61 (15)	44 (10.9)	105 (26)
11–20	20 (4.95)	48 (11.88)	68 (16.8)
21–30	44 (10.9)	63 (15.59)	107 (26.5)
31–40	24 (5.9)	34 (8.4)	58 (14.4)
41–50	16 (3.96)	14 (3.46)	30 (7.4)
51–60	10 (2.5)	9 (2.2)	19(4.7)
≥ 60	10 (2.5)	7 (1.7)	17 (4.2)
Total	185 (45.8)	219 (54.2)	404 (100)

The majority of male patients prescribed corticosteroids were under 10 years old (15%), while the largest number of female patients between the ages of 21 and 30 were prescribed corticosteroids (15.6%). Female patients aged 60 or older made up the minimum number of patients who took corticosteroids (1.7%). See Table 2.

**Table 2:** Age vs. potency of TCS prescribed at dermatology OPD, Injibara General Hospital, 2024.

Age (years)	Very high potent	High potent	Moderately potent	Low potent	Total	%
0–10	3	32	30	42	107	24.6
11–20	20	26	24	0	70	16
21–30	46	25	43	3	117	26.9
31–40	19	22	21	1	63	14.5
41–50	12	9	10	4	35	8
51–60	10	5	7	0	22	5
≥ 60	10	4	5	1	20	4.6
Total	120	123	140	51	434	100

### Pattern of skin diseases

**Figure 1:**
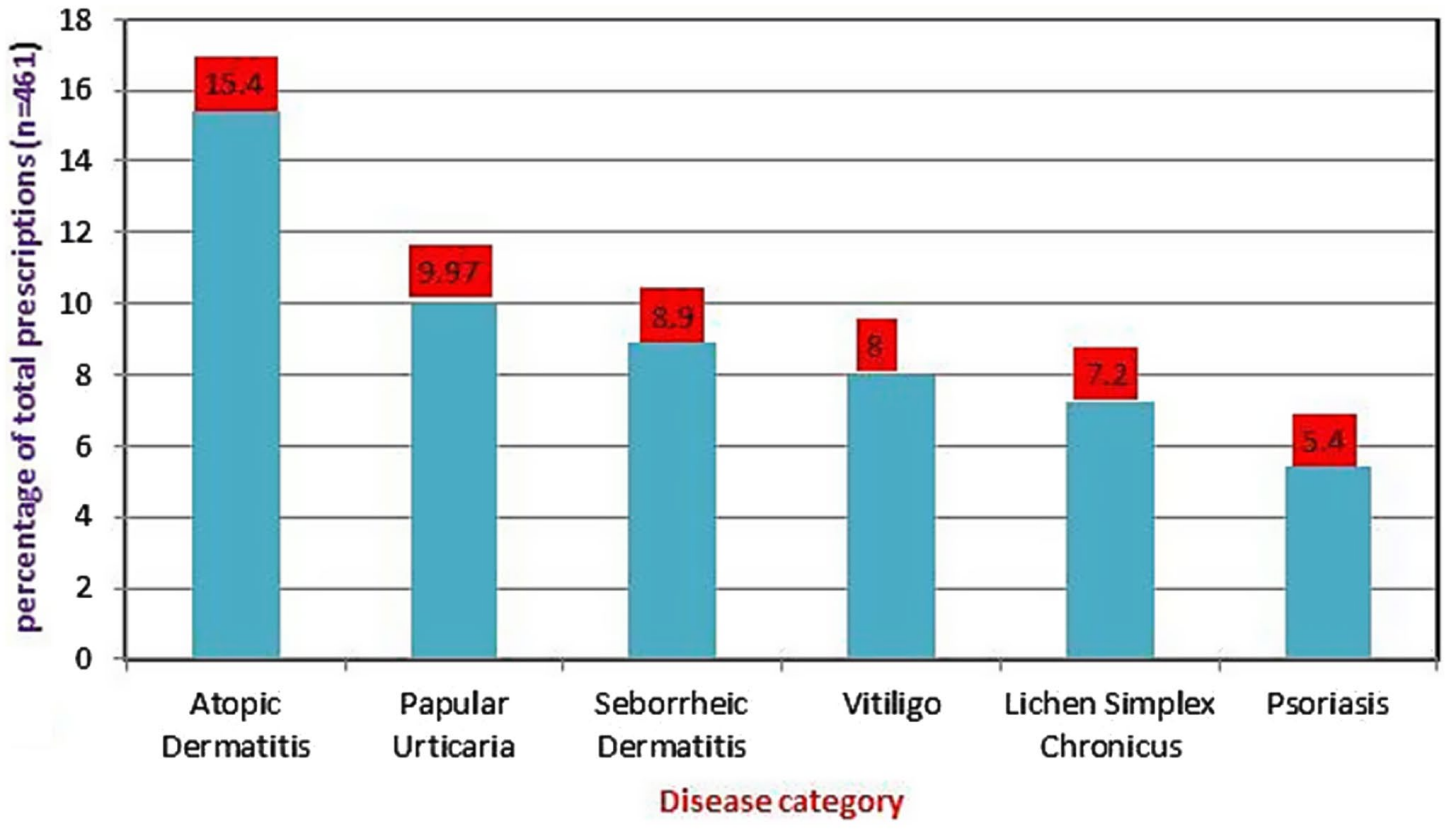
Disease category for which corticosteroids were mostly prescribed at dermatology outpatient department, Injibara General Hospital, 2024.

Based on disease distribution, out of 404 patients, a total of 421 diagnosed diseases were issued corticosteroids. Atopic dermatitis (15.4%) was the most common skin disorder, followed by papular urticaria (9.97%), seborrheic dermatitis (8.9%), and vitiligo (8%) for which corticosteroids were indicated. See Figure 1.

### Prescribing practice of corticosteroids

**Figure 2:**
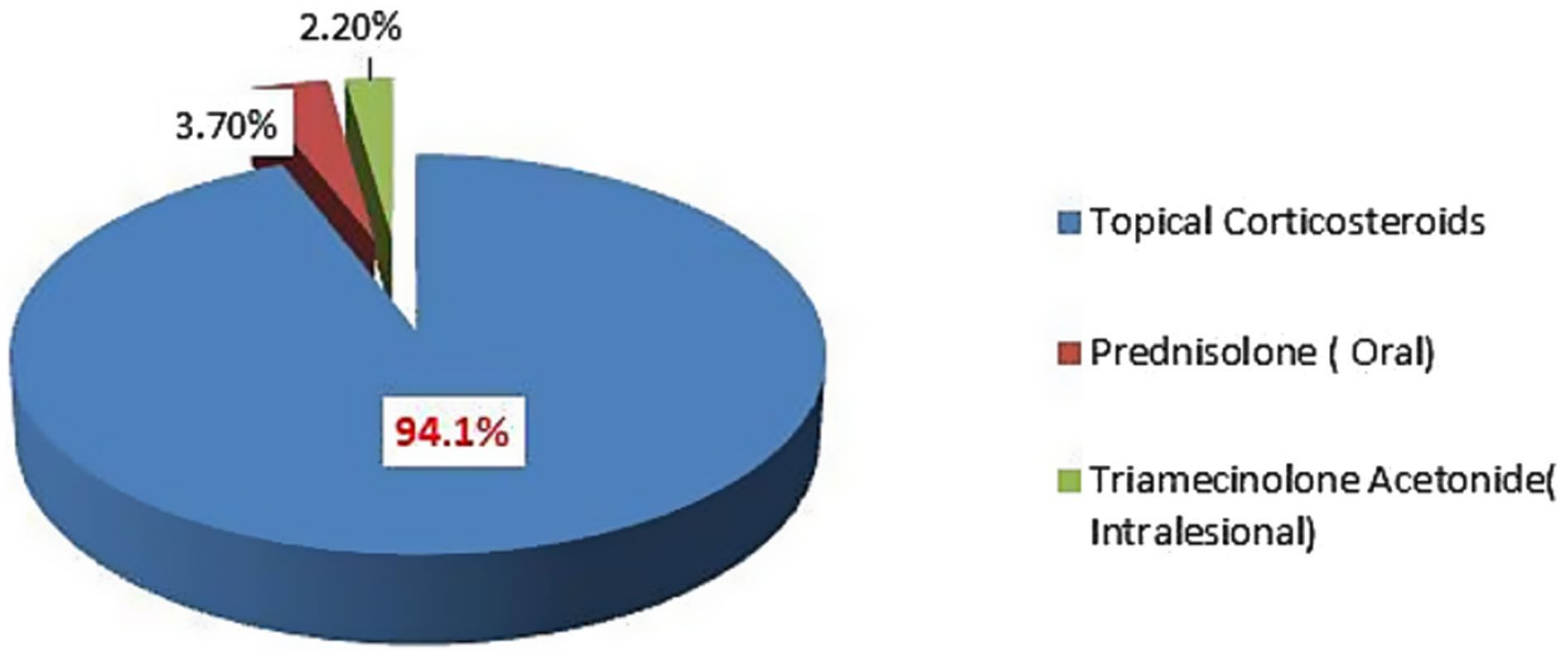
Details of corticosteroids prescribed at dermatology outpatient department, Injibara General Hospital, 2024.

Topical corticosteroids were the most commonly prescribed corticosteroids, accounting for 434 (94.1%), followed by oral 17 (3.7%) and intra-lesional corticosteroids 10 (2.2%). See Figure 2.

Of 434 TCS, mometasone furoate was the most commonly prescribed (34.6%), followed by clobetasol propionate (26.7%). Ointments (53%) followed by creams (40.6%) were the most common formulations of TCS issued. The percentage of corticosteroids prescribed by generic name was 82.4%. We found that the percentage of TCS prescriptions in FDC and compounding was 4.4 and 1.2%, respectively. See Table 3.

**Table 3:** Details of topical corticosteroids prescribed at dermatology OPD, Injibara General Hospital, 2024.

	Nomenclature		Prescription item			Formulations			Total, *N* = 434 (%)
	Generic (*N* = 353)	Brand (*N* = 81)	Single (*N* = 410)	FDC (*N* = 19)	Compounding (*N* = 5)	Ointment (*N* = 230)	Cream (*N* = 176)	Lotion (*N* = 28)	
Mometasone Furoate 0.1%	122	28	148	–	2	65	69	16	150(34.56%)
Clobetasole propionate 0.05%	84	32	116	–	–	111	4	1	116(25.57%)
Betamethasone valerate 0.1%	52	3	52	3	–	43	9	–	55(12.67%)
Hydrocrtisone acetate 1%	48	3	46	3	2	–	51	–	51(11.75%)
Floucinolone acetonide 0.025%	34	–	33	–	1	–	34	–	34(7.8%)
Betamethasone Dipropionate 0.05%	13	15	15	13	–	11	9	11	28(6.45%)
Total	353	81	410	19	5	230	176	28	

The number of corticosteroids per prescription varied from 1 to 4. Out of 404 corticosteroid-treated patients, 356 (77.2%) patients were prescribed only one corticosteroid, while 105 (22.8%) patients were given more than one corticosteroid, like oral corticosteroid along with topical corticosteroids or more than one TCS. Among patients requiring corticosteroids for their skin disorder, the average number of corticosteroids per encounter was found to be 1.14. See Table 4.

**Table 4:** Number of corticosteroids prescribed at dermatology OPD, Injibara General Hospital, 2024.

No. of corticosteroids per prescription	Frequency (*N* = 461)	Percentage (%)
1	356	77.2
2	80	17.4
3	21	4.6
4	4	0.8
Total	461	100

**Figure 3:**
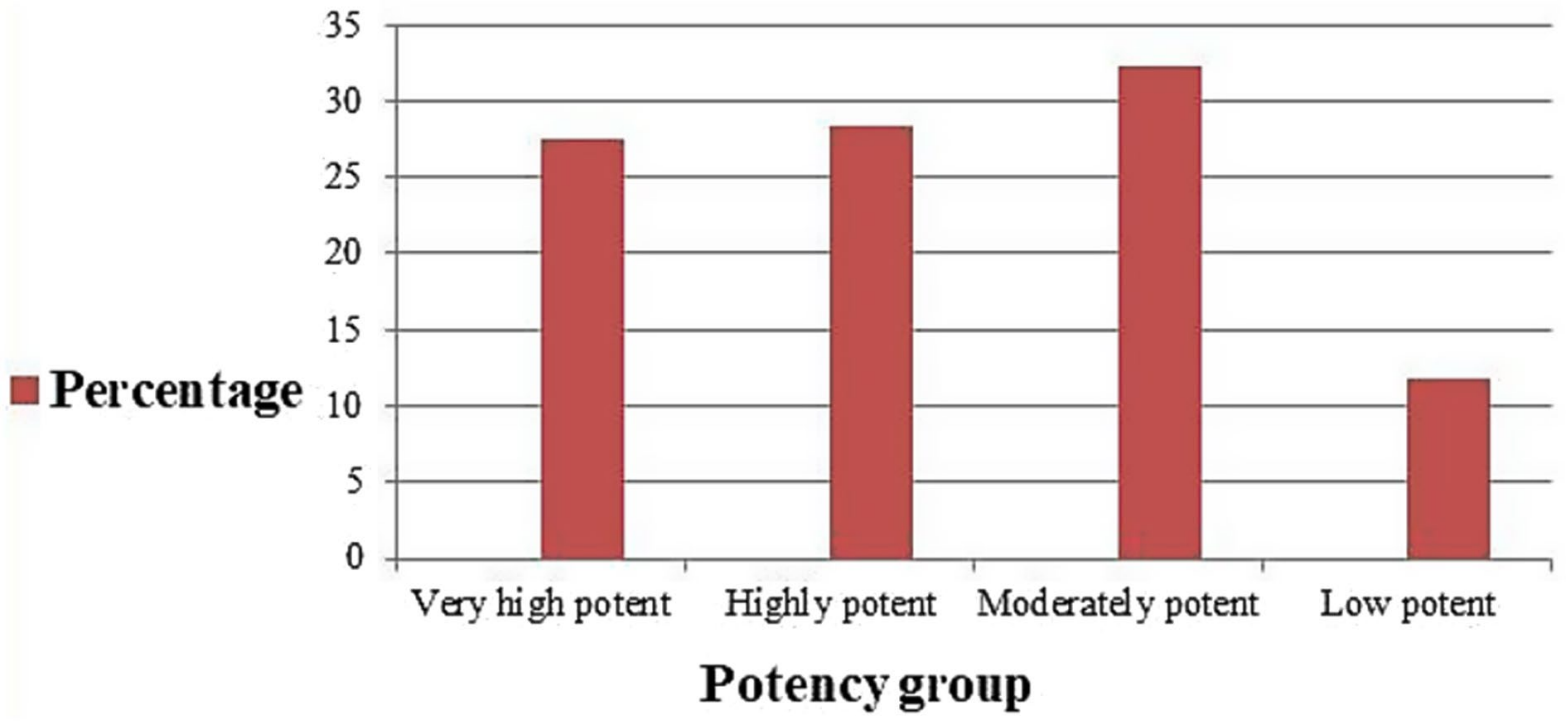
Potency class of TCS prescribed at dermatology outpatient department, Injibara General Hospital, 2024.

In the present study, moderate potency TCS were most commonly prescribed (32.3%), followed by high potency (28.3%), and super high potency (27.6%), respectively. See Figure 3.

## Discussion

The topic of prescribing patterns for dermatological drugs has been the subject of numerous studies. Some of these have shown that potent TCS are being overused or used inappropriately.

In this study, out of all the corticosteroids prescribed (461), more than half (247, or 53.6%) of the corticosteroids were prescribed to female patients, consistent with study findings from ALERT Hospital (11), Indian rural tertiary teaching hospital (12), and Kathmandu University Hospital (13). The high number of females using corticosteroids for dermatological purposes may be attributed to their increased awareness of skin health. Furthermore, women use cosmetics at a higher rate than males do, which may have particularly predisposed them to a variety of skin conditions.

Analysis of the patient data revealed that inflammatory skin conditions, mainly dermatitis, were the most common diagnosis where corticosteroids were prescribed. This is consistent with study findings from a tertiary care teaching hospital of tribal region of central-south India (14). However, a study from Kathmandu University Hospital, Dhulikhe, revealed that urticaria was the most common dermatologic disorder where patients were prescribed corticosteroids (13). Different epidemiologic patterns of skin problems in various locations could account for this difference.

Out of all the corticosteroids prescribed in this study, 82.4% were prescribed by generic names. The habit of generic drug prescription is better compared to study results from ALERT hospital, where only 2.5% of corticosteroids were prescribed by generic names (11). Similarly, our study is far better than the study findings from North Palestine, where all TCS were prescribed by their brand names (5). However, this is lower than the study findings from a tertiary care teaching hospital in a tribal region of central-south India, where 88.9% of corticosteroids were prescribed by their generic names (14). Using brand names for prescribing may sometimes create dispensing errors (5). In our setting, similar appearing brands have different ingredients and are more costly than generic ones. Therefore, using their generic names when prescribing medications could reduce costs and prescription errors.

Among all the TCS, mometasone furoate 0.1% was the most commonly prescribed drug (34.56%), followed by clobetasole propionate 0.05% (25.57%), a very high potent TCS. A study from ALERT hospital and Tribhuvan University Teaching Hospital demonstrated that betamethasone dipropionate and clobetasole propionate was the most commonly prescribed TCS (11, 15). Similarly, our result is different from studies conducted in tertiary care teaching hospital of the tribal region of Central-South India and North Palestine, where betamethasone valerate 0.1% was the most commonly prescribed TCS, at 55.34 and 22%, respectively (5, 14). A study from India Tertiary Care Hospital (16) and Tertiary Care Hospital, Malabar, Kerala (17), also demonstrated that clobetasole propionate was the most commonly prescribed TCS. The pattern of prescription may be influenced by the availability of the preparation in the hospital pharmacy, disease patterns, and the choice of the dermatologist.

Our study demonstrated that, FDC accounted for 4.4% of total TCS prescriptions with betamethasone dipropionate-Miconazole and betamethasone valerate-fusidic acid combinations. This is lower than the study findings from where 27.7% of prescriptions were FDC with beclomethasone-clotrimazole and beclomethasone-fusidic acid combinations (18). Similarly, the rate of FDC prescription was 54.45% from the tertiary teaching hospital of Mewat (19). This difference in magnitude and type of FDC may be determined by the availability and experience of dermatologists with FDCs.

Extemporaneous compounding prescriptions of TCS accounted for 1.2% of prescriptions in the present study. This is consistent with the study findings from Palestine, where the prevalence of extemporaneous compounding is 1.5% (20). This figure aligns with a systematic review that demonstrated that the prevalence of extemporaneous compounding was less than 5% (21). In the present study, most extemporaneous compounding prescriptions were made for melasma and psoriasis, which is consistent with study the finding from ALERT hospital (22). Extemporaneous compounding services are only found in regional city, Bahirdar, and patients were sent with their prescriptions to get the medication.

In the present study, ointments followed by creams were the most common formulations of TCS issued. This is similar to study findings with ALERT hospital, where ointments followed by creams were the most commonly dispensed formulations in dermatology outpatients (11). In our study, TCS were most commonly prescribed for eczematous disorders. As a result, ointments are more suitable for dry skin and are more effective than other formulations.

A majority of the patients in this study used TCS, followed by systemic corticosteroids. A very small number of the patients used corticosteroids by both the topical and systemic routes. Oral prednisolone and intralesional triamecinolone acetonide were the only systemic steroids prescribed. Our finding is consistent with several other studies where topical corticosteroids were preferred over systemic corticosteroids for dermatologic disorders (13, 23–25). This might be due to the effectiveness of topical corticosteroids in many non-infective skin disorders and the fact that they are safer to use for acute skin diseases (23, 26).

In the present study, TCS from the four potency category, i.e., low, moderate, high, and very high potent TCS, were prescribed. Nonetheless, moderate potency (32.3%) TCS were found to be the most commonly prescribed TCS followed by high potency TCS (28.3%). The aforementioned result was in line with research conducted in America, which showed that the most often prescribed TCSs were moderately potent and high potent (27). From ALERT hospital, high potency followed by very high potent TCS were most commonly prescribed (11). Out of all the topical steroids prescribed, 117 (26.9%) were prescribed within the age group 21–30 years, followed by 0–10 years 107(24.6%). From Indian rural tertiary teaching hospital, 94.36% of TCS prescriptions belonged to very high potent groups (12). Most of the very high potent and moderately potent topical steroids, together, are the most commonly prescribed class of TCS for ages 0–10 years (9.6%). Our study is in agreement with the study findings from ALERT Hospital (7). A study from Kathmandu University Hospital, Dhulikhel, showed that low-potency TCS were used most commonly, followed by high-potency TCS (13). In this study, there is a tendency to prescribe high and very high potency TCS in the dermatology outpatient department. The prescription of very high potent corticosteroids should be limited when possible. Long and excessive use may carry the risk of suppression of the hypothalamic–pituitary–adrenal axis as well as local adverse effects (28).

### Strength of the study

This is the first study in Ethiopia to evaluate prescription patterns of corticosteroids at outpatient dermatology department.

### Limitations of the study

The study was done in a single health facility. As a result, the study is not representative of the general population. The study was also retrospective. Future researchers are thus encouraged to conduct a prospective study.

## Conclusion and recommendations

From this study, it can be concluded that atopic dermatitis is the most common skin disease observed in the studied dermatology outpatient where the corticosteroid was indicated. TCS were the most commonly prescribed medications and ointment was the most common formulation prescribed. Use of the drugs mostly by generic name is a welcome initiative toward rationality. It was also found out that mometasone furoate and clobetasole propionate were the two most commonly prescribed agents, which are moderate or high potency and very high potency TCS, respectively. Usage of extemporaneous compounding and FDC was low. Prescribing practice of high potent and very high potent topical corticosteroids was found to be considerably high.

Future researchers are advised to undertake multicenter studies with a high number of study participants to provide a national picture of corticosteroid prescriptions, as this study was limited by the fact that it was conducted in a single health facility and only involved a small number of patients. Implementation of training programs for dermatologists and other healthcare providers to ensure they are updated on the latest guidelines and best practices for corticosteroid use is encouraged. Establishing a robust system for monitoring patients on corticosteroids to minimize potential side effects and improve safety is recommended. Development and dissemination of clear guidelines tailored to the local context to facilitate appropriate prescribing practices is encouraged.

## Data Availability

The original contributions presented in the study are included in the article/supplementary material, further inquiries can be directed to the corresponding author.
